# Genomic Features and Tissue Expression Profiles of the Tyrosinase Gene Family in the Chinese Soft-Shelled Turtle (*Pelodiscus sinensis*)

**DOI:** 10.3390/genes16070834

**Published:** 2025-07-17

**Authors:** Yanchao Liu, Pan Liu, Tong Ren, Yang Gao, Ziman Wang, Junxian Zhu, Chen Chen, Liqin Ji, Xiaoyou Hong, Xiaoli Liu, Chengqing Wei, Xinping Zhu, Zhangjie Chu, Wei Li

**Affiliations:** 1 School of Fishery, Zhejiang Ocean University, Zhoushan 316000, China; liuyanchao204@163.com (Y.L.);; 2 Key Laboratory of Tropical & Subtropical Fishery Resource Application & Cultivation of Ministry of Agriculture and Rural Affairs, Pearl River Fisheries Research Institute, Chinese Academy of Fishery Sciences, Guangzhou 510380, Chinazjzhujunxian@prfri.ac.cn (J.Z.); hxy@prfri.ac.cn (X.H.); liuxl@prfri.ac.cn (X.L.);; 3 Science and Technology Research Center of China Customs, Beijing 100026, China; 4 School of Marine and Fisheries, Guangdong Eco-Engineering Polytechnic, Guangzhou 510520, China

**Keywords:** *Pelodiscus sinensis*, body color, TYR gene family, genomic features, expression analysis

## Abstract

In farmed animals, body color is not only an ecological trait but also an important trait that influences the commercial value of the animals. Melanin plays an important role in the formation of body color in animals, while the tyrosinase (TYR) gene family is a group of key enzymes that regulate melanogenesis. The Chinese soft-shelled turtle (*Pelodiscus sinensis*) is one of the most important reptiles in freshwater aquaculture. However, the potential role of the TYR gene family in the body color formation of *P. sinensis* remains unclear. This study aimed to investigate the expression and conservation of the TYR gene family in relation to body color variation in *P. sinensis*. Three core members of this gene family were identified from the *P. sinensis* genome. Following identification, the genomic features were analyzed. They shared similar physicochemical properties and conserved domains. Chromosome mapping showed that the three genes of *P. sinensis* were all located on the autosomes, while phylogenetic and collinearity analysis suggested that the protein functions of the three genes in the studied species were highly conserved. Amino acid sequence alignment indicated high conservation among the three TYR gene family proteins (TYR, TYRP1, and DCT) in multiple critical regions, particularly in their hydrophobic N-/C-terminal regions and cysteine/histidine-rich conserved domains. The qRT-PCR revealed that the *TYR* and *DCT* genes were highly expressed in the eyes of individuals with different body colors. The expression levels of *TYR* and *TYRP1* genes in the skin were significantly higher in dark-colored individuals than in light-colored ones (*p* < 0.05). These results will lay the groundwork for functional studies and breeding programs targeting color traits in aquaculture.

## 1. Introduction

Body color is one of the most important phenotypic characteristics in animals and plays a crucial role in identification, mate selection, camouflage, and warning [1]. In farmed animals, body color is not only an ecological trait but also an important trait that affects the commercial value of these animals [2]. *P. sinensis* is a commercially important freshwater turtle, prized for its nutritional and medicinal properties, as well as its culinary appeal [3,4]. Body color serves as a key selective breeding trait that influences market demand [5], as evidenced by the commercial success of the Yongzhang golden turtle (GS-01-011-2018), a selectively bred ornamental yellow-bodied variety. Therefore, cultivating novel color variants to enhance ornamental value requires deeper research into *P. sinensis*’ body color formation mechanisms.

Melanin is one of the most important pigments in the process of body color formation and can be divided into two types: eumelanin and pheomelanin [6]. The melanogenesis process begins with the TYR-catalyzed oxidation of tyrosine and DOPA to DOPAquinone. Subsequent reactions diverge based on cellular conditions. In the presence of cysteine (Cys) or glutathione (GSH), DOPAquinone converts into pheomelanin. When neither of them is present, DOPAquinone performs cyclization to produce DOPAchrome, which either undergoes spontaneous decarboxylation to yield TYR-catalyzed eumelanin or tautomerization to generate eumelanin by tyrosinase-related proteins (TYRP1 and DCT) activity [7]. It can be seen that TYR gene family members play key roles in melanogenesis and animal body color formation.

TYR gene family belongs to the copper-containing metal enzyme family, which is a class of genes encoding key enzymes in melanin synthesis and which contains three main members: *TYR*, *TYRP1*, and *DCT* [8]. Yu et al. identified five *TYR* variants in the Muchuan Black-Boned Chicken (*Gallus gallus domesticus*), and the expressions of all variants were significantly different in black and white feather bulbs (*p* < 0.05) [9]. By establishing a synchronized Tyrosinase transport system, Nakamura et al. demonstrated that *TYRP1* is a positive regulator that promotes melanogenesis, which has the function of promoting Tyrosinase targeting to melanosomes [10]. Braasch et al. studied the method of knockdown of the *TYRP1* gene in zebrafish (*Danio rerio*), which caused it to produce pheomelanin instead of eumelanin [11]. In addition, a study suggested that changes in the expression levels of *DCT* may be closely related to changes in the body color of Rainbow trout (*Oncorhynchus mykiss*) [12]. Zhang et al. found that compared to wild-type turtle (AWT), the Yongzhang golden turtle (YGT) exhibited significantly reduced melanin content but markedly increased carotenoid levels. Furthermore, the downregulation of genes *bc01* and *bc02*, which are negatively correlated with carotenoid content, may be associated with the development of yellow pigmentation in YGT [13]. Additionally, Wang et al. identified key pigmentation regulators in *P. sinensis* by comparing two distinct color morphs using mRNA-seq and miRNA-seq, including mRNAs (*MITF*, *GPNMB*, *KIT*) and miRNAs (miR-138, miR-383-3p, miR-1388b-5p) [14]. However, functional studies of the role of the TYR gene family in the body color formation of *P. sinensis* have not been reported.

In this study, TYR gene family members were identified and analyzed based on the genome of *P. sinensis*. The differential expressions of the identified genes in the individuals with different body colors were detected by means of quantitative reverse transcription PCR (qRT-PCR). This study will provide basic data for further research on the evolutionary and functional mechanisms of the TYR gene family in *P. sinensis.*

## 2. Materials and Methods

### 2.1. Genome Identification and Physicochemical Analysis of the TYR Gene Family

First, we retrieved the genomic and proteomic data of *P. sinensis* (GCF_000230535.1) from the National Center for Biotechnology Information (NCBI) database, including genome sequences, protein sequences, and annotation files. Subsequently, the Hidden Markov Model (HMM) data of the TYR gene (PF00264) were obtained from the Pfam Protein Family Database. To identify members of the TYR gene family in the *P. sinensis* genome, we then employed HMMER 3.0 software [15]. Following this identification, we assessed the candidate sequences and removed the sequence redundancy using the SMART database, with all parameters set to the default values [16]. The molecular weight (kDa), theoretical isoelectric point (pI), instability coefficient, fat index, and total mean hydrophilic coefficient (GRAVY) of the TYR proteins were calculated using ProtParam (https://web.expasy.org/protparam/, accessed on 14 July 2025) [17]. Finally, the subcellular localizations of the TYR proteins were analyzed using Cell-PLocr 2.0 [18].

### 2.2. Chromosome Scaffold Mapping and Gene Structure Analysis of the TYR Gene Family

Based on the genome annotation file, chromosome scaffold mapping and gene structure visualization of the filtered TYR gene family members were performed using the TBtools 2.2 [19].

### 2.3. Phylogeny and Collinearity Analysis of the TYR Gene Family

The amino acid sequences of three core TYR gene family members (*TYR*, *TYRP1*, and *DCT*) from the 14 vertebrate species were retrieved from the NCBI database. The 14 vertebrate species were *P. sinensis*, *D*. *rerio*, *Oryzias latipes*, *Bufo gargarizans*, *Xenopus tropicalis*, *Homo sapiens*, *Mus musculus*, *Zootoca vivipara*, *Thamnophis elegans*, *Coturnix japonica*, *Gallus gallus*, *Chelonia mydas*, *Mauremys mutica*, and *Mauremys reevesii*. Following sequence acquisition, multiple-sequence alignment was performed using MUSCLE 5.1[20] to ensure accurate comparisons across species. The phylogenetic tree is a commonly used analytical tool in molecular biology for identifying gene family members and analyzing phylogenetic relationships among species [21]. The species’ evolutionary trees were constructed using the maximum likelihood method with the Jones–Taylor–Thornton (JTT) substitution model and gamma-distributed (G) rate heterogeneity in MEGA 7 [22]. Node support was assessed with 1000 bootstrap replicates, and the final tree was visualized using tvBOT (https://chiplot.online/tvbot.html, accessed on 14 July 2025)[23].

### 2.4. Conserved Motifs and Structural Domain Analysis of the TYR Gene Family

Conserved motifs in TYR proteins were predicted using the MEME Suite database, with a maximum of 9 motifs allowed [24]. The structural domains were then annotated with the NCBI Conserved Domain tool [25], with all filter parameters set to default values. Finally, both results were visualized using TBtools software [19].

### 2.5. Homology Alignment and Structural Analysis of the TYR Proteins

Eight representative species spanning mammals, birds, amphibians, reptiles, and fish were selected for comparative analysis with *P. sinensis*, including *H. sapiens*, *G. gallus*, *X. tropicalis*, *Z. vivipara*, *O. latipes*, *C. mydas*, *M. mutica*, and *M. reevesii*. Sequence alignment of the TYR gene family was performed using BioEdit 7.2 [26]. Protein secondary structures were predicted using SOPMA (https://npsa.lyon.inserm.fr/cgi-bin/npsa_automat.pl?page=/NPSA/npsa_sopma.html, accessed on 14 July 2025)[27], while 3D modeling was conducted with SWISS-MODEL [28].

### 2.6. Transcription Factor Analysis

The 2 kb upstream promoter regions of TYR genes were extracted from *P. sinensis* genome annotation files. Then, transcription factors were predicted using the JASPAR database Scan tool (https://jaspar.elixir.no/tools/, accessed on 14 July 2025), with a screening threshold set at 0.8 [29].

### 2.7. Quantitative Reverse Transcription PCR Analysis of the TYR Gene Family

#### 2.7.1. Sample Collection and Ethical Declaration

**Figure 1:**
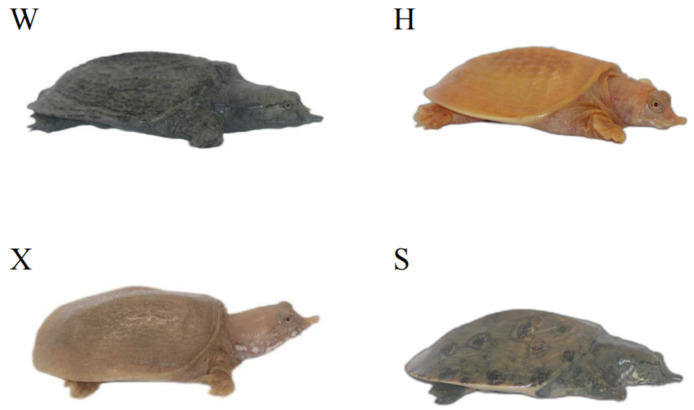
Phenotypic photographs of *P. sinensis* individuals with four different body colors. The four individuals are referred to as the Qingxi black strain (**W**), Yongzhang golden strain (**H**), albino strain (**X**), and Huangsha strain (**S**).

To explore the functions of TYR gene family members, qRT-PCR was utilized to measure their relative expression levels across the tissues of four *P. sinensis* individuals with different body colors (Figure 1): the Qingxi black strain has dark-green skin and black eyes, and was provided by Zhejiang Qingxi Soft-shelled turtle Industry Co. (Huzhou, China); the Yongzhang golden strain has golden-yellow skin and red eyes, and was provided by the Fuping County Jingtao soft-shelled turtle farm (Baoding, China); the albino strain has pinkish-white skin and red eyes, and was provided by the Liwan District Jiying Breeding Farm (Guangzhou, China); the Huangsha strain has olive-green skin and black eyes, and was provided by the Qinzhou City Lin Jisheng Huangsha soft-shelled turtle farm (Qinzhou, China). All *P. sinensis* individuals used in this study were healthy one-year-old specimens (mean weight: 42.92 ± 4.08 g). Different tissue samples were obtained by following the sample collection method described by Wang et al. [30]. We collected ten types of tissue, namely heart, liver, spleen, lung, kidney, intestine, brain, muscle, eye and skin tissues. The collected samples were promptly preserved in liquid nitrogen for RNA isolation. All experimental methods used for the studied species were performed in accordance with the animal husbandry regulations of the Pearl River Fisheries Research Institute, located in Guangzhou, China.

#### 2.7.2. RNA Extraction and Quantitative Reverse Transcription PCR

Total RNA was extracted from the different tissues according to the instructions for TRlzol Reagent (Life Technologies, Carlsbad, CA, USA). A total of 20 mg of tissue and 1 mL of TRIzol were added into an RNase-free EP tube, homogenized thoroughly and incubated at RT (10 min). Then, 0.2 mL of chloroform was added and the mixture was vortexed vigorously, incubated at RT (5 min), and then centrifuged at 12,000 rpm (4 °C) for 15 min. The upper aqueous phase was carefully transferred to a new tube and an equal volume of isopropanol was added. This mixture was mixed by vortexing and incubated at RT (10 min). It was then centrifuged at 12,000 rpm (4 °C) for 10 min and the supernatant was discarded. The white pellet at the bottom contained RN; this pellet was washed with 1 mL of 75% ethanol, centrifuged at 12,000 rpm (4 °C) for 5 min, and the supernatant was discarded. The washing step was repeated once. After discarding the supernatant, the EP tube was opened and air-dried (5 min) and the pellet was dissolved in 20 μL of RNase-Free H_2_O. The RNA concentration and purity were measured using NanoDrop 2000 (Thermo Fisher Scientific, Wilmington, DE, USA). RNA integrity was assessed using an Agilent Bioanalyzer 2100 system (Agilent Technologies, Santa Clara,CA, USA). The first strand of cDNA was synthesized using the reverse transcription kit from Takara (Beijing, China). Total RNA was mixed with RT Primer Mix, incubated at 70 °C (10 min), chilled on ice (3 min), and then combined with 2 μL of 5 × M-MLV Buffer, 0.5 μL of dNTP Mixture, 0.25 μL of RNase inhibitor, and 0.5 μL of RTase MLV (RNase H**^—^**). The reaction was conducted at 42 °C (60 min) and then the mixture was incubated at 70 °C (15 min). As a highly sensitive and specific technique for measuring the relative expression levels of genes through the fluorescent detection of amplified DNA during PCR cycles [31], qRT-PCR was carried out using iTaq Universal SYBR Green Supermix (Bio-Rad, Hercules, CA, USA) on ten tissue types from four *P*. *sinensis* color morphs, with three biological replicates per tissue and three technical replicates each. Primers were designed based on the available nucleotide sequences of the TYR gene family from the NCBI (Table 1). Since the expression pattern of the *Ef1a* gene was consistent across all tissues of *P. sinensis*, it was used as a reference gene to calculate the relative expression levels of the target genes [32]. Primer specificity was verified by NCBI BLAST(https://blast.ncbi.nlm.nih.gov/Blast.cgi, accessed on 14 July 2025) and confirmed by a single peak in the melting curve (Supplementary Figure S1). Subsequently, qRT-PCR standard curves demonstrated high linearity (R^2^ = 0.994–0.999), with slopes (−3.16 to −3.34) corresponding to amplification efficiencies of 99–107% (Supplementary Figure S2). Gene expression levels were quantified using the 2^−ΔΔCt^ method [33]. Differential expression was analyzed by one-way ANOVA using GraphPad Prism 8.0.2 [34], with results presented as the mean ± SEM of triplicate experiments (statistical significance threshold: *p* < 0.05) [35].

## 3. Results

### 3.1. Identification and Physicochemical Characterization of the TYR Gene Family Members

Three TYR gene family members were identified in the genome of *P. sinensis: TYR*, *TYRP1*, and *DCT*. The results of the physicochemical characterization were as follows: The number of amino acids ranged from 526 to 535. The molecular weight ranged from 59.8 to 60.1 kD. The isoelectric points ranged from 5.35 to 6.47. The instability index ranged from 42.55 to 51.51. The aliphatic index ranged from 74.01 to 76.00. The average hydropathicity ranged from −0.289 to −0.326. Subcellular localization revealed that the three TYR gene family members were all localized on the plasma membrane (Table 2).

### 3.2. Chromosome Scaffold Mapping and Gene Structure of the TYR Gene Family Members

**Figure 2:**
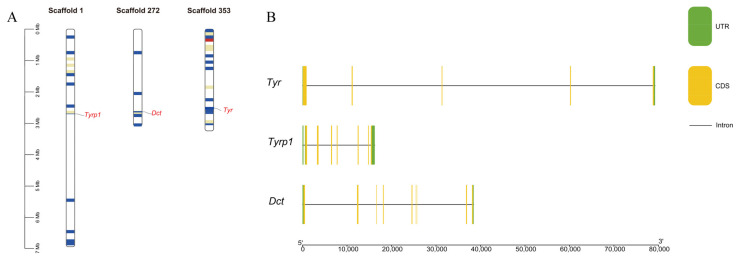
(**A**) Chromosome scaffold mapping and (**B**) gene structure analysis of the TYR gene family members in *P. sinensis*. In (**A**), the colored lines indicate the gene density, with red lines representing regions of higher density. The gene density is defined as the number of genes within a 50 kb genome. In (**B**), the green boxes, yellow boxes, and black lines represent the non-untranslated regions, exons, and introns, respectively.

Our chromosome localization analysis showed that the three screened TYR gene family members were distributed on chromosome scaffold 1, chromosome scaffold 272, and chromosome scaffold 353, respectively (Figure 2A). In the gene structure analysis, there were differences in the gene length, coding sequences, and introns of the three TYR gene family members. The *TYR* gene had five coding sequences and four introns. The *TYRP1* gene had seven coding sequences and seven introns. Finally, the *DCT* gene had eight coding sequences and seven introns (Figure 2B).

### 3.3. Phylogenetic Relationships of the TYR Gene Family Members in 14 Vertebrate Species

**Figure 3:**
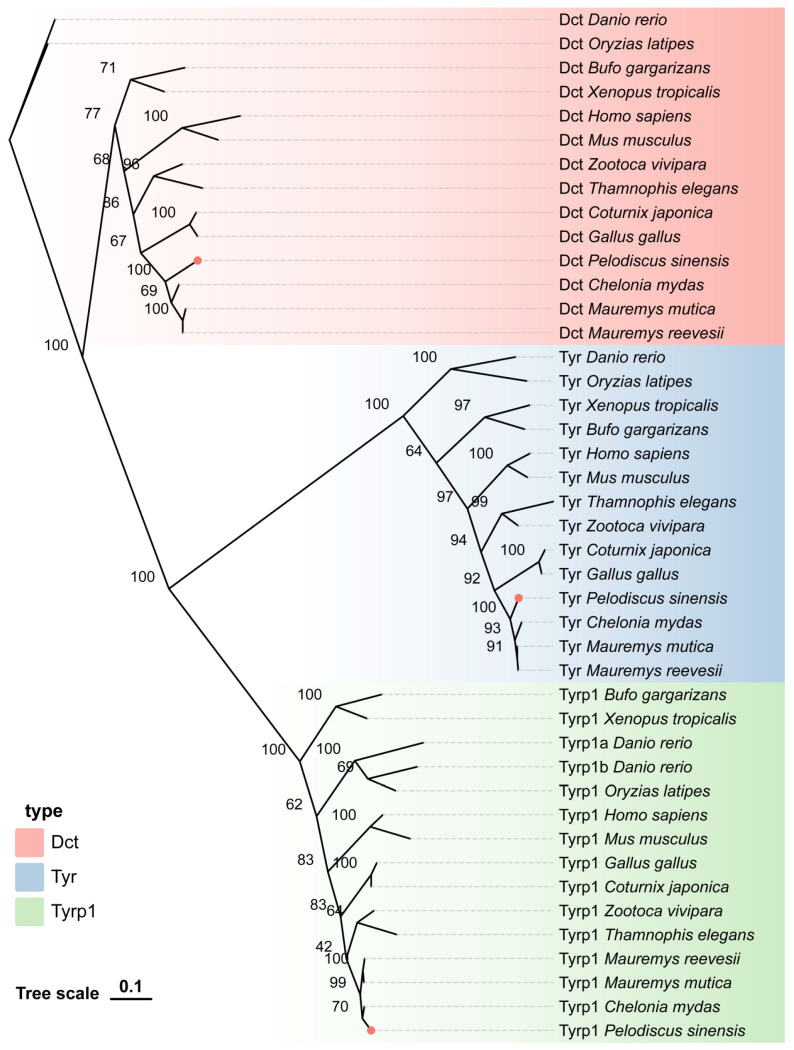
Phylogenetic tree of the relationship of the TYR gene family members in *P. sinensis* with other vertebrates.

We constructed a phylogenetic tree to investigate the phylogenetic relationships of the TYR gene family in *P. sinensis*. The three screened genes from the TYR gene family were divided into three subfamilies. Based on the topology of the phylogenetic tree, it can be seen that the *TYR* and *DCT* genes of *P. sinensis* exhibited the smallest genetic distance with turtles, birds, and reptiles, followed by mammals and amphibians, and the farthest genetic distance with fish. However, the *TYRP1* gene exhibited the closest genetic distance with turtles, reptiles, and birds, followed by mammals and fish, and the farthest genetic distance with amphibians (Figure 3).

### 3.4. Collinearity Analysis of the TYR Gene Family Members

**Figure 4:**
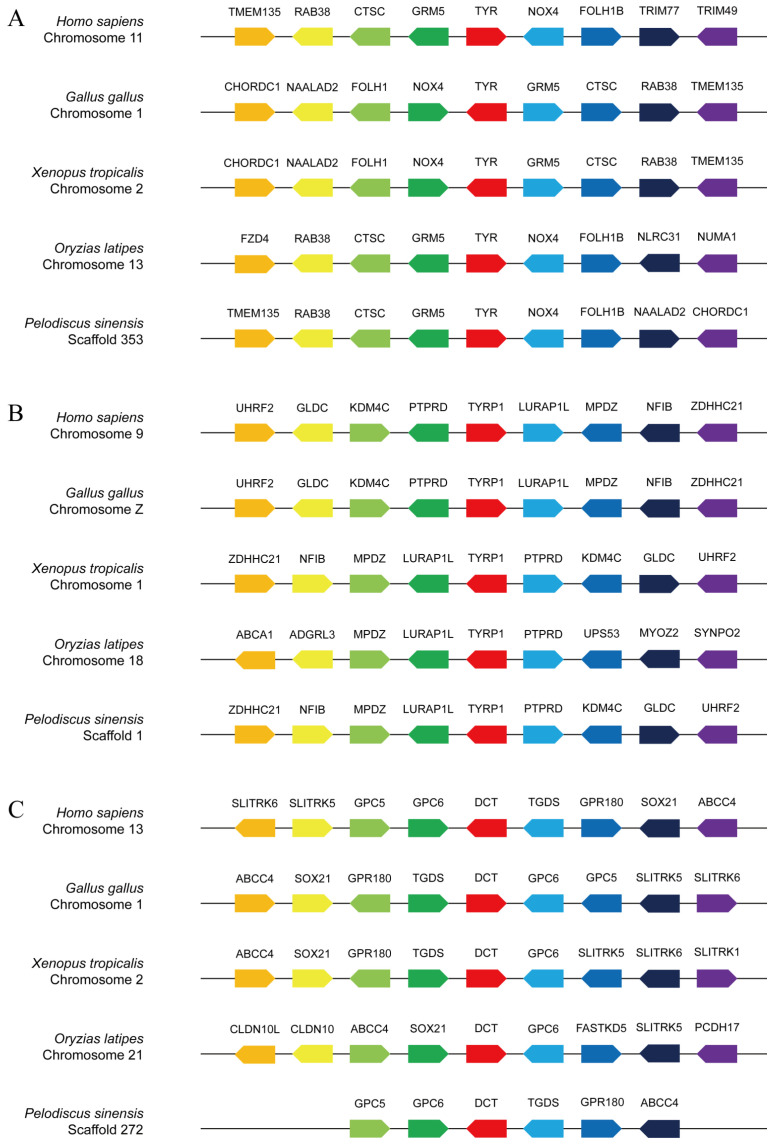
Collinear analyses of TYR (**A**), TYRP1 (**B**), and DCT (**C**) between *P. sinensis* and other animals. The direction of the arrow indicates the transcription direction, and each solid line represents a chromosome scaffold.

A collinear analysis was conducted across a number of species, including *H. sapiens*, *G. gallus*, *X. tropicalis*, *O. latipes*, and *P. sinensis* (Figure 4). The *TYR* genes of *H. sapiens*, *G. gallus*, *X. tropicalis*, and *O. latipes* were located on chromosomes 11, 1, 2, and 13, respectively (Figure 4A). The *TYRP1* genes of *H. sapiens*, *X. tropicalis*, and *O. latipes* were located on chromosomes 9, 1, and 18, respectively (Figure 4B). It is worth noting that the *TYRP1* gene of *G. gallus* was located on sex chromosome Z. The *DCT* genes of *H. sapiens*, *G. gallus*, *X. tropicalis*, and *O. latipes* were located on chromosomes 13, 1, 2, and 21, respectively (Figure 4C). Based on the chromosome positioning results, it could be observed that the *TYR* and *DCT* genes of *G. gallus* and *X. tropicalis* were located on the same chromosomes. Furthermore, we also found that the *TYR* genes of *H.sapiens*, *G. gallus*, *X. tropicalis*, and *P. sinensis* were located on the same gene module, TMEM135-RAB38-CTSC-GRM5-TYR-NOX4. This segment was highly conserved during evolution. *O*. *latipes* only had the conserved fragment RAB38-CTSC-GRM5-TYR-NOX4, while TMEM135 was replaced with FZD4 (Figure 4A). The *TYRP1* genes of *H.sapiens*, *G. gallus*, *X. tropicalis*, and *P. sinensi* were located on the same gene module, UHRF2-GLDC-KDM4C-PTPRD-TYRP1-LURAP1L-MPDZ-NFIB-ZDHHC21. *O. latipes* only had the conserved fragment PTPRD-TYRP1-LURAP1L-MPDZ, while KDM4C was replaced with UPS53, and NFIB was replaced with ADGRL3 (Figure 4B). The *DCT* genes of *H.sapiens*, *G. gallus*, and *P. sinensis* were located on the same gene module, GPC5-GPC6-DCT-TGDS-GPR180. However, similar conserved gene modules were not found in the *DCT* genes of *X. tropicalis* and *O. latipes* (Figure 4C).

### 3.5. Conserved Motifs and Structural Domain Analysis of the TYR Gene Family Members

**Figure 5:**
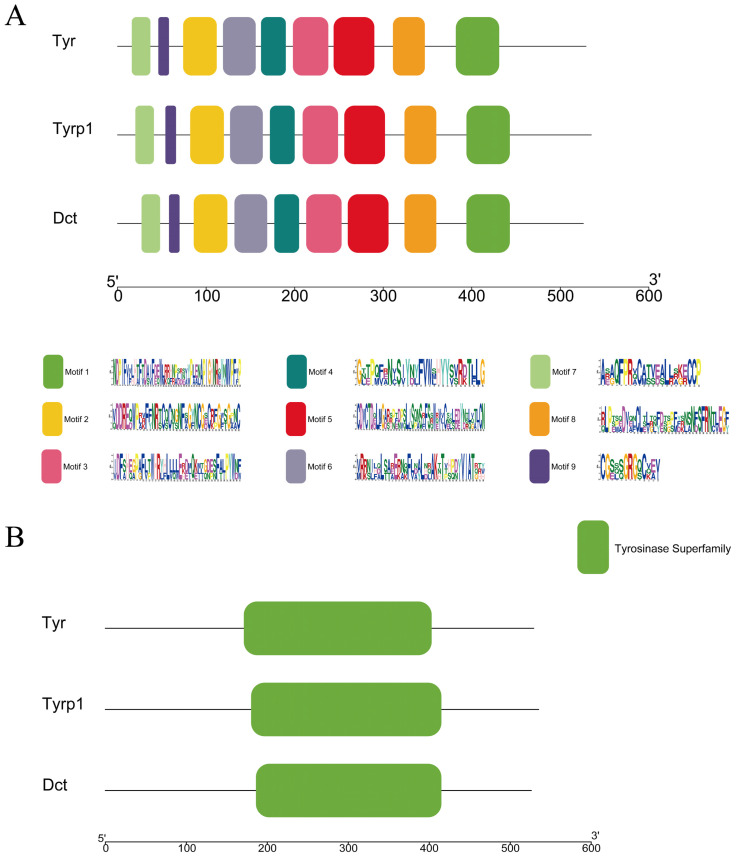
(**A**) Conserved motifs and (**B**) structural domain analysis of the TYR gene family members in *P. sinensis*. Motifs 1–9 are highlighted by distinct colored boxes in (**A**). The Tyrosinase Superfamily domain is indicated by the green box in (**B**).

Through amino acid sequence conservation region prediction, nine of the most conserved motifs were obtained (Figure 5A), which were all in the *TYR*, *TYRP1*, and *DCT* gene amino acid sequence. In addition, the three TYR gene family proteins contained the same domain, namely the Tyrosinase Superfamily (Figure 5B). Our results showed that these protein motifs were highly conserved.

### 3.6. Amino Acid Sequence Alignment and Protein Structure Prediction of the TYR Gene Family Members

**Figure 6:**
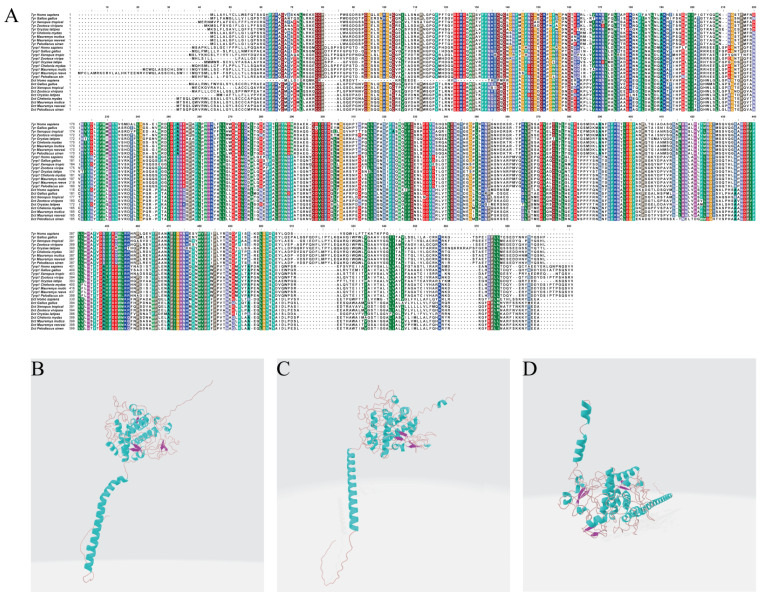
(**A**) Amino acid sequence alignment of the TYR gene family members in *P. sinensis* with other vertebrates. Structural analysis of the (**B**) TYR, (**C**) TYRP1, and (**D**) DCT proteins in *P. sinensis*. In (**B**–**D**), blue spirals represent α-helices, purple arrows represent β-turns, and brown lines represent random coils.

The results of the amino acid sequence alignment indicated that the three TYR gene family proteins (TYR, TYRP1, and DCT) were highly conserved in multiple critical regions across the nine species. All sequences featured hydrophobic amino acids in their N- and C-terminal segments. Moreover, they all contained multiple conserved Cys and histidine (His) residues. The sequence alignment results for TYR protein showed that the similarity between *P. sinensis* and other Testudines species is above 97%, while the lowest similarity is with fish, at only 76.3%. The sequence alignment results for TYRP1 protein indicated that the similarity between *P. sinensis* and other Testudines species is above 91%, while the lowest similarity is with amphibians, at only 82.2%. The sequence alignment results for DCT protein revealed that the similarity between *P. sinensis* and other Testudines species is above 91%, while the lowest similarity is with fish, at only 76% (Figure 6A). The results of our protein 3D structure model prediction of the three TYR gene family members showed that the tertiary structure of TYR, TYRP1, and DCT proteins mainly consisted of α-helices, β-turns, random coils, and extended strands (Figure 6B–D). Specifically, the proportions of TYR protein were 28.36%, 1.89%, 59.17%, and 10.59%. The proportions of TYRP1 protein were 30.65%, 1.87%, 56.45%, and 11.03%, while those of DCT protein were 29.85%, 3.80%, 52.85%, and 13.50%.

### 3.7. Transcription Factor Prediction of the TYR Gene Family Members

**Figure 7:**
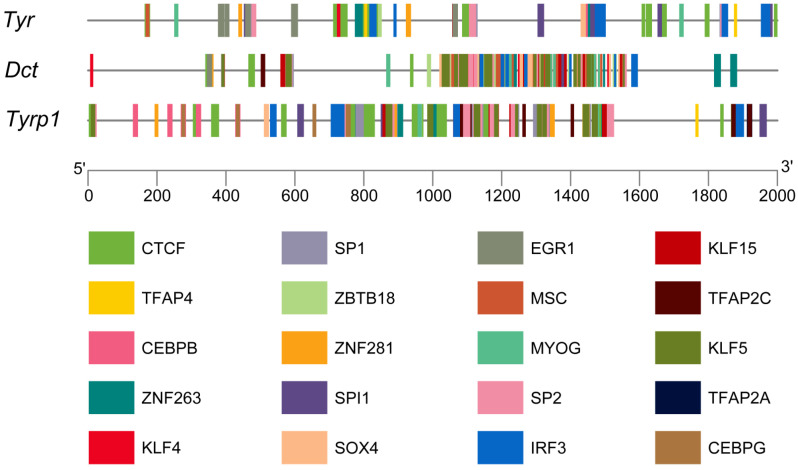
Analysis of the promoters of the TYR gene family members in *P. sinensis*. Different-colored rectangles indicate different transcription factors.

In this study, 750, 707, and 763 types of transcription factors were predicted in the promoter regions of the *TYR*, *TYRP1*, and *DCT* genes, respectively. The top 20 transcription factors were selected for visualization and analysis. CTCF accounted for the largest number of transcription factors. The transcription factors of the TFAP and KLF family were the most diverse. Among these, TFAP2A, TFAP2C, and KLF4 are associated with the development and function of melanogenesis (Figure 7).

### 3.8. Expression Profiling of the TYR Gene Family Members in Four Kinds of P. sinensis

**Figure 8:**
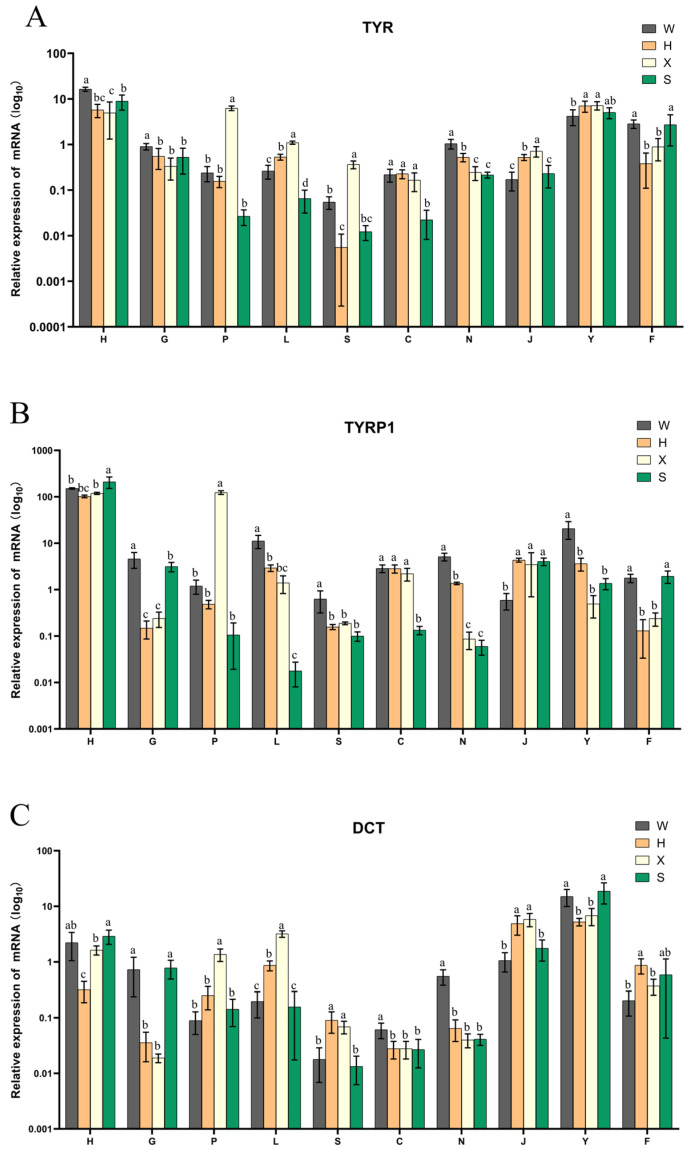
Differential expressions of (**A**) *TYR*, (**B**) *TYRP1*, and (**C**) *DCT* in four kinds of *P. sinensis*. The four individuals are referred to as the Qingxi black strain (W), Yongzhang golden strain (H), albino strain (X), and Huangsha strain (S). The ten tissues are coded as heart (H), liver (G), spleen (P), lung (L), kidney (S), intestine (C), brain (N), muscle (J), eyes (Y), and skin (F) tissues. In the bar chart, means labeled with different lowercase letters indicate a significant difference (*p* < 0.05), whereas means labeled with the same lowercase letters are not significantly different.

The qRT-PCR results showed that all three members of the TYR gene family were expressed in all tissues of different body colors (Figure 8). *TYR* and *TYRP1* had significantly higher expression levels in the skin of dark-colored individuals than in light-colored individuals (*p* < 0.05). The *TYR* and *DCT* genes were highly expressed in the eyes of individuals with different body colors. The expression of all three genes in kidney and intestine tissues was relatively low.

## 4. Discussion

Melanin is one of the most important pigments involved in the formation of body coloration, serving functions such as light absorption, photoprotection, and skin pigmentation [36]. The TYR gene family is widely found in eukaryotes and prokaryotes and plays an important role in melanogenesis [37]. This study identified three major members of the TYR gene family in the genome of *P. sinensis* with similar physicochemical properties. Subcellular localization revealed that these were all localized on the plasma membrane, and that the three TYR gene family proteins all had a conserved domain, namely the Tyrosinase Superfamily. The results of our gene structure analysis showed that the TYR gene family members differ in gene length, coding sequence, and introns, suggesting that they may be functionally diverse. Phylogenetic analyses can be used to identify the evolutionary relationships of different species. Judging from the phylogenetic tree, the *TYR* and *DCT* genes of *P. sinensis* are the closest to those of turtles, birds, and reptiles and the farthest from fish. Unlike the above two genes, the *TYRP1* gene exhibited a longer genetic distance from amphibians compared with fish. The results of the collinearity analysis and amino acid sequence alignment are consistent with the phylogenetic analysis, suggesting that the protein functions of the three genes in these species are highly conserved. In the amino acid sequence alignment, we found several conserved amino acid residues, including Cys and His. Cys, an amino acid containing a sulfhydryl group, plays a critical role in protein folding and structural stability through the formation of disulfide bonds via oxidation between two cysteine residues [38]. Cys also plays an important role in melanogenesis; under the action of Cys, DOPAquinone is converted into pheomelanin [7]. The catalytic activity of tyrosinase, the key enzyme regulating melanogenesis, is modulated by histidine residues. Through site-directed mutagenesis, Nakamura et al. showed that replacing any of the seven histidine residues that coordinate copper ions with asparagine in *Escherichia coli* expressing recombinant *Aspergillus oryzae* tyrosinase completely abolished its enzymatic activity [39]. Also using site-directed mutagenesis, Hyangsoone’s team identified three histidine residues at the CuA site as essential for human tyrosinase activity. These histidine residues likely participate directly in copper coordination, thereby catalyzing the hydroxylation of L-tyrosine [40]. The TYR gene family plays a crucial role in melanin synthesis, and its evolutionary conservation is essential for maintaining melanocyte function [41].

Our chromosome positioning results showed that the three TYR gene family members in all the investigated species were located on the autosomes, except the *TYRP1* in *G. gallus*, which was located on the Z chromosome. Birds have a ZW sex determination system, where males are ZZ and females are ZW [42]. Sexual dimorphism in plumage color is common, with males often exhibiting brighter and more decorative feathers than females, likely due to sexual selection favoring ornamental traits in males [43]. Additionally, the presence of two Z chromosomes in males may enhance the expression of Z-linked color genes, such as *TYRP1*, which may contribute to green head feathers in male ducks, as identified through transcriptome analysis [44]. Meanwhile, the TYR gene family members located on autosomes also play a crucial role in melanogenesis and body color formation in animals. Zhang et al. cloned and analyzed the *TYR* and *TYRP1* genes from both normal and albino yellow catfish (*Tachysurus fulvidraco*). They found that the mRNA expression levels of both genes exhibited significant tissue specificity in two phenotypes, with higher expression observed in normal individuals compared to albino individuals [45]. Melina et al. analyzed the expression levels of *TYR* in the skin of Llamas (*Lama glama*) with different coat colors using qPCR. The results showed that the expression of *TYR* in white llamas was significantly lower than that in dark-pigmented phenotypes (*p* < 0.05) [46].

This study also identified several transcription factors that are associated with melanin synthesis in the promoter regions of the three TYR gene family members of *P. sinensis*, such as TFAP2A, TFAP2C and KLF4. TFAP2A and TFAP2C are members of the TFAP2 transcription factor family, which plays a crucial role in regulating melanocyte differentiation and melanoma phenotypes [47]. O’Brien et al. found that the melanocytes of zebrafish embryos which underwent knockdown as a part of TFAP2A treatment were reduced and showed abnormal differentiation and migration [48]. In addition, Seberg et al. found that TFAP2A/TFAP2B double-conditional mutation-treated mouse embryos had significantly fewer melanocytes than the controls. They also analyzed TFAP2A-deficient zebrafish tissue and mouse melanocytes and found that the expression of a subset of pigmentation-associated genes was TFAP2A-dependent, including that of the *DCT* gene [49]. Hong et al. found that the mRNA and protein levels of KLF4 were more highly expressed in black-skinned sheep than in sheep with white skin. Moreover, the overexpression of KLF4 significantly increased the expression of the *TYR* gene [50]. As can be seen from the above results, the three main members of the TYR gene family of *P. sinensis* may participate in the formation of body color through the transcriptional regulation of TFAP2A, TFAP2C and KLF4.

The qRT-PCR results for different tissues with different body colors of *P. sinensis* showed that the expression levels of the *TYR* and *TYRP1* genes are significantly higher in the skin tissue of dark-colored individuals than in light-colored individuals (*p* < 0.05). As the first effective barrier between living organisms and the environment, the skin has the role of preventing pathogens from invading, resisting chemical and physical attacks, and preventing water loss [51]. Skin pigmentation is the most important photoprotective factor of skin, in which melanin has an ultraviolet absorption effect, as well as antioxidant and free radical scavenging characteristics. A study showed that *TYR* is a key gene for the regulation of Bali cattle’s (*Bos javanicus domesticus*) fur color. Its expression differed significantly between standard Bali cattle and albinos (*p* < 0.05) [52]. The research of Gong et al. showed that the expression of *TYRP1* in the normal Russian sturgeon (*Acipenser gueldenstaedtii*) was significantly higher than in the albino phenotype [53]. As can be seen from the above results, *TYR* and *TYRP1* play important roles in melanogenesis. At the same time, their high expression levels affect pigmentation and skin protection. The *TYR* and *DCT* genes were highly expressed in the eye tissues of individuals with different body colors. TYR is a key rate-limiting enzyme in melanin production. Mutations in the *TYR* gene will cause reduced or even absent forms of hair, skin, and eye pigmentation, which is known as ocular skin albinism (OCA) [54]. Andrson et al. found that nm2798-mutant mice exhibited the phenomenon of iris pigment dispersion, while the nm2798 mutation was a missense mutation in the *DCT* [55]. These results indicate that the *TYR* and *DCT* genes play important roles in the process of iris pigmentation. The *TYR*, *TYRP1* and *DCT* genes show differential expression patterns in the spleen, liver, and kidney tissues among the four different-colored strains of *P. sinensis*. These three organs serve as crucial immune regulatory organs in animals [56,57,58]. The current study has demonstrated that the TYR gene family plays a role in immune regulation in animals [59]. Furthermore, some studies have shown differences in the immune regulatory capacity between different-colored individuals within the same species, such as the Threespine stickleback (*Gasterosteus aculeatus*) [60] and Chinese mitten crab (*Eriocheir sinensis*) [61]. Therefore, we speculate that the differential expression of TYR gene family members in these three organs may indicate differences in immune regulatory capacities among different-colored individuals of *P. sinensis*. The above findings demonstrate that the TYR gene family is associated with body color formation and plays a crucial role in melanogenesis. While it has been shown that its function in vertebrate pigmentation regulation is evolutionarily conserved [37,62,63], information regarding the TYR gene family in the *P. sinensis* remains scarce. Our results demonstrate that the TYR gene family regulates body color variation in *P. sinensis*. These findings will provide a theoretical foundation for understanding the molecular mechanisms of pigmentation and practical data for targeted breeding programs of color traits in aquaculture. However, our current findings are limited to transcriptional evidence of TYR gene family regulation, with additional protein-level validation and expanded sample sizes needed to strengthen the conclusions in future studies.

## 5. Conclusions

In this study, three major members of the TYR gene family were identified. Their function in *P. sinensis* pigmentation regulation is evolutionarily conserved. The tissue expression profiles of *TYR* and *TYRP1* suggest their functional contribution to body color variation in *P. sinensis*. These findings will provide fundamental data for research on the functional mechanisms of color characteristics and selective breeding of ornamental traits in aquaculture. Additionally, the immunomodulatory functions of the TYR gene family will be a key focus of our future investigations.

## Figures and Tables

**Table 1:** Primer sequences for qRT-PCR analysis.

Gene	Primer Sequence (5′-3′)	Amplicon Length (bp)	Tm (°C)
*TYR*	F: GTTTACCCAGCAGCCAATGC R: TGGAAGGAGTCAACTGGGTCT	152	60
*TYRP1*	F: CTCCACACTGCTCCTTACAC R: CTATTACGGCTACACATTGACC	176	58
*DCT*	F: GAGCTGCCAGTGTACAGGAAA R: TCACATAGTCTGGGTGCGTG	194	60
*Ef1a*	F: ACTCGTCCAACTGACAAGCCTC R: CACGGCGAACATCTTTCACAG	253	60

**Table 2:** Information on the TYR gene family members.

Gene	Number of Amino Acids	Molecular Weight	Theoretical pI	Instability Index	Aliphatic Index	Grand Average of Hydropathicity	Subcellular Localization
*TYR*	529	60,152.08	5.56	51.51	74.90	−0.289	Plasma membrane
*TYRP1*	535	60,157.45	5.35	49.75	76.00	−0.331	Plasma membrane
*DCT*	526	59,858.20	6.47	42.55	74.01	−0.326	Plasma membrane

## Data Availability

The data presented in this study are available on request from the author. Email: liuyanchao204@163.com.

## Supplementary Materials

The following supporting information can be downloaded at: https://www.mdpi.com/article/10.3390/genes16070834/s1, Figure S1. Melting curve analysis of *TYR* (A), *TYRP1* (B), *DCT* (C), and *Ef1a* (D); Figure S2. Amplification efficiency analysis of *TYR* (A), *TYRP1* (B), *DCT* (C), and *Ef1a* (D); Table S1: Transcription factors regulating *TYR*, *TYRP1*, and *DCT*.
