# Supplementary material for: Genomic Features and Tissue Expression Profiles of the Tyrosinase Gene Family in the Chinese Soft-Shelled Turtle (Pelodiscus sinensis)
Source: Genes (Basel). 2025 Jul 17;16(7):834. doi: 10.3390/genes16070834 (PMC12294924; doi:10.3390/genes16070834)
Supplement: Supplementary file 1 [file genes-16-00834-s001.zip › genes-3732519-supplementary.pdf]

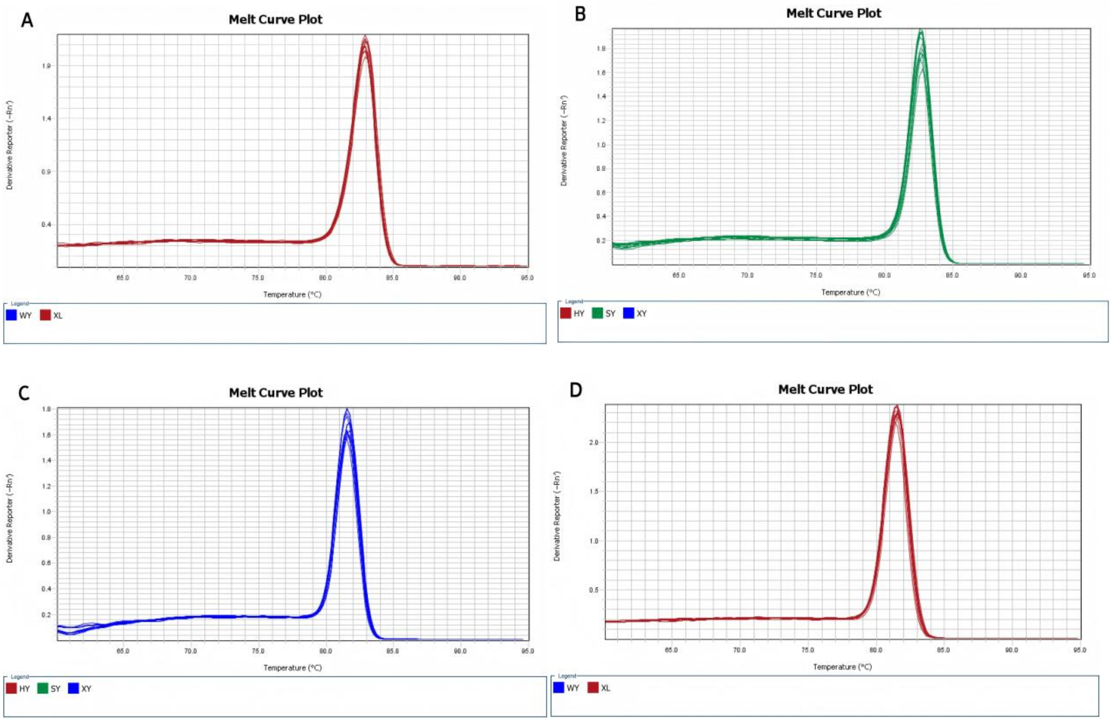

Supplementary Figure S1. Melting curve analysis of *TYR* (A), *TYRP1* (B), *DCT* (C), and *Ef1a* (D).

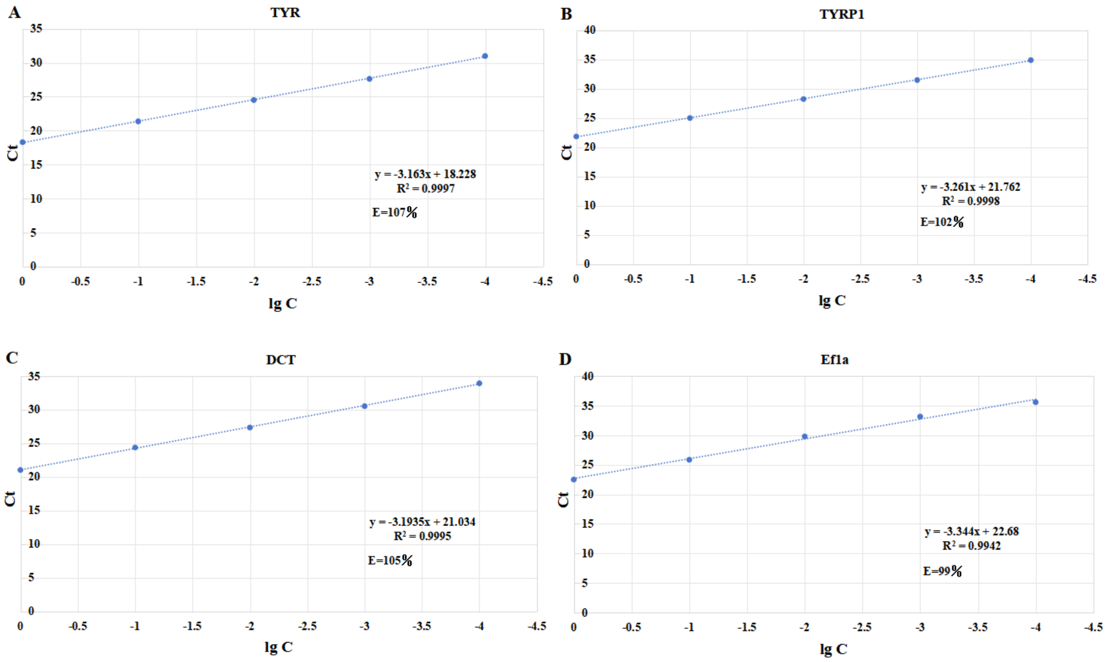

Supplementary Figure S2. Amplification efficiency analysis of *TYR* (A), *TYRP1* (B), *DCT* (C), and *Ef1a* (D).
